# Failure Predictor Factors of Conservative Treatment in Pediatric Forearm Fractures

**DOI:** 10.1155/2018/5930106

**Published:** 2018-07-12

**Authors:** G. Maccagnano, A. Notarnicola, V. Pesce, S. Tafuri, S. Mudoni, V. Nappi, B. Moretti

**Affiliations:** ^1^Orthopaedics Unit, Department of Basic Medical Science, Neuroscience and Sensory Organs, Faculty of Medicine and Surgery, University of Bari, General Hospital, Bari, Italy; ^2^Department of Biomedical Sciences and Human Oncology, Faculty of Medicine and Surgery, University of Bari, General Hospital, Italy; ^3^Faculty of Medicine and Surgery, University of Bari, General Hospital, Italy

## Abstract

The aim of this study is to evaluate the predictive efficacy of the radiographic parameters and the relationship between the radiographic results and the clinical data. We carried out a retrospective study analyzing the data of 225 pediatric patients with forearm fractures treated conservatively. Two orthopaedists examined 4 different radiographic parameters. They compared CI and radial translation parameters at T0, in terms of indication of type of treatment and predictive efficacy. Afterwards, the two orthopaedists analyzed X-rays performed at T1, evaluating radiographic results according to radial shortening and angle parameters. From the analysis of the CI measured by Observer 1, 135 patients out of 225 had retrospective indication to conservative treatment; the frequency of failure was 18/135 (13.3%). Observer 2 indicated conservative treatment in 144 patients out of 225 and the proportion of failure was 21/144 (14.6%). As regards the radial translation, Observer 1 reported a frequency of failure of 78/225 (34.7%) and Observer 2 reported 75/222 (33.8%). Furthermore the authors detected a deficit of pronosupination for the patients considered to have failure according to radiographic results. The authors defined the greater reliability of CI with respect to the radial translation parameter and the direct relationship between radiographic failure and clinical-functional data.

## 1. Introduction

Forearm fractures are among the most common lesions in pediatric patients [1, 2]. 20-30% of the cases involve distal metaphysis [3, 4].

81% of forearm fractures happen to children over the age of 5, with the peak of incidence between 10 and 12 years of age in females and 12 and 14 in males. The mechanism of trauma is directly related to falling on the palm of the hands [5].

Conservative treatment consists in carrying out a reduction on manoeuvre and long arm cast [5, 6]; in 85% of cases this achieves a good clinical-functional result [7].

Osteosynthesis is indicated in cases of open fractures and physis fractures or when conservative treatment fails [8].

As regards conservative treatment the most common complication is loss of the reduction [9, 10]. For this reason recent literature reports an increase in the use of k-wires as a first treatment [11, 12].

This renders the study of radiographic parameters even more important in understanding the predictive efficacy of conservative treatment [13]. The therapeutic strategy adopted depends principally on radiographic parameters even though there is no clear scientific evidence to support this given that in literature the vast majority of works analyze either radiographic indices linked to the type of fracture or the parameters linked to the manoeuvre reduction but not both together.

The aim of this study is to evaluate the predictive efficacy of the radiographic parameters, the interobserver reproducibility, and the relationship between the radiographic results and the clinical data.

## 2. Material and Methods

We carried out a cohort retrospective study analyzing the clinical-radiographic records of 225 pediatric patients with diaphyseal forearm fractures according to Orthopaedic Trauma Association Classification (22 D/2.1, D/4.1, and D/5.1). The subjects, aged between 2 and 15, had been treated conservatively in our hospital in the period 2010-2014 with an average follow-up of 18 months.

The conservative treatment consisted in the reduction manoeuvre of the fracture and cast moulding. The casts were applied above the elbow. The elbow was immobilized at 90° degrees. The casts were composed of Cellona Plaster of Paris Bandages.

The reduction manoeuvre and the cast application were performed by two trauma surgeons (B.M. and V.P.) with at least 10 years of experience in the pediatric field.

Inclusion criteria for the study were

- monoosseous forearm fracture,
- biosseous forearm fracture,
- age range of 2-15.

Exclusion criteria were

- open or pathological forearm fracture,
- physeal fracture,
- patients who did not complete follow-up and those for whom there was no complete radiographic documentation.

The study population was divided into 3 groups: Group 1 < 5 years, Group 2 with range of 5-10 years, and Group 3 > 10 years. The data collected regarded sex, age, side of fractures (left/right), and type.

The radiographic parameters examined were

- Cast Index (CI), described for the first time by Chess et al. in 1994 [14],
- radial translation, which evaluates the translation of distal radius segment with respect to proximal segment [15],
- shortening of radius with respect to ulna [16],
- angle between bone segments in lateral view [17].

The CI evaluates the efficacy of the reduction manoeuvre and the geometry of the cast. It calculates the ratio between the distance of internal margins of the cast in lateral view (x) and anteroposterior view (y) at the point of fracture. In the literature a high risk of loss of reduction when CI is ≥0.84 is reported [14].

The radial translation allows the distinguishing of 4 different groups: the first without any translation; the second with translation of distal radius segment <50%; the third > 50%;the fourth when there is no contact between the segments. Mani found that in 60% of patients in Groups III and IV there was a secondary failure of reduction manoeuvre [15].

The shortening of radius with respect to ulna is responsible for pronosupination deficits if the value reaches a pathological range > 5mm [16].

The angle subtended between bone segments in lateral view allows us to define a pronosupination limit if the value is >15° in patients aged under 10 years and >10° in children aged over 10 years [17, 18].

The measurements of radiographic parameters, described previously, were calculated on digital X-rays which were performed at a distance of 100cm in 2 projections (anteroposterior and lateral views). ROMAN v 1.7 software was used to calculate 4 parameters after the calibration of the system in order to convert pixel into mm.

Two orthopaedists with an experience greater than 10 years performed measurements blindly and independently.

All the patients were followed up at 1 year (T_1_) after the trauma. Follow-up examinations included measurements of forearm range of motion for both sides using a goniometer. The position of the arm was adducted and elbow flexed at 90°.

The two orthopaedists indicated retrospectively the type of treatment (surgical or conservative) according to CI and radial translation values measured at T_0_ by emergency X-ray. As defined in literature, in the presence of the values of CI and radial translation, respectively, ≥0.84 and >50% of bone diameter, the two observers suggested surgical treatment.

Afterwards, the two orthopaedists analyzed X-rays performed at T_1_, evaluating radiographic results according to radial shortening and angle parameters. As reported in literature, an angle value greater than 15° in children under 10 years and greater than 10° in patients over 10 years and a radial shortening value greater than 5mm are indicative of poor result and pronosupination deficit. Furthermore, the two surgeons compared poor radiographic data to pronosupination range of motion.

For each patient enrolled, a data collection form structured in different sections was compiled:

- Anagraphic variables (name, surname, age), type, and side of fracture
- variables measured by two observers: CI and radial translation at T_0_ and radius shortening and inclination of segments in lateral view at T_0_ and T_1_
- Treatment performed.

The compiled forms were put into a database using the fileMaker Pro software and analyzed with STATA software.

The qualitative variables (sex, age groups, side, indication of surgical treatment based on CI and radial translation, and patients with poor outcome based on inclination and radial shortening parameters) were described in percentages; the chi-square test was used to evaluate the differences between the percentages.

The quantitative variables (age, CI, radial shortening, and inclination) were expressed in mean values with standard deviation. The Student t-test for paired samples was used to evaluate the differences between the mean values measured by the two observers.

To evaluate the level of agreement between the two measuring processes (Observers 1 and 2) in relation to the indication of surgical treatment according to CI and radial translation at T_0_ and indication for failure based on inclination and radial shortening, the K-test agreement was used.

The Bland Altman graph, calculating the values of r and Z, was developed to quantify any eventual differences between the measurements of the two observers of CI at T_0_ and radial shortening and inclination at T_0_ and T_1_.

For each test used, the p value <0.05 was considered significant.

## 3. Results

In the study 225 patients were enrolled; of these 183 (81.3%) were male and 42 (18.7%) female. The average age was 8.4±3.5 years (range 2-15); in particular 36 (16%) were under 5, 111 (49.3%) were between 5 and 10, and 78 (34.7%) were over 10.

The affected side was right in 93 (41.3%) and left in 132 (58.7%). Among the 225 recruitment patients, 84 (37.3%) presented monoosseous radius fracture, 9 (4%) monoosseous ulna fracture, and 132 (58,7%) biosseous fracture.

**Figure 1:**
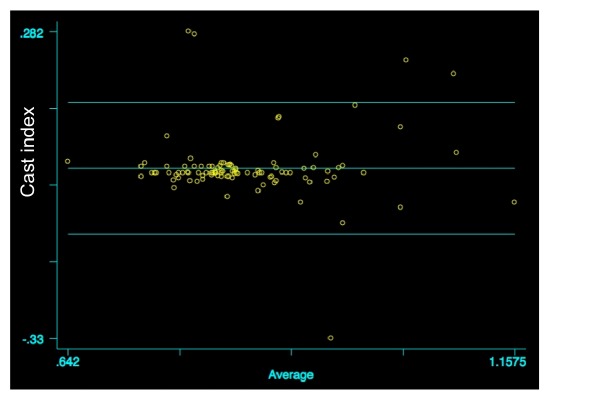
Comparison of CI values between the two measurements, Bland Altman graph (r=0.027; p=0.79).

The mean value of CI, measured by Observer 1, was 0.85±0.09 and 0.84±0.9, by Observer 2 (t=1.41; p=0.08). The Bland Altman test, used to compare the measurements, did not give evidence of any statistically significant differences (r=0.027; p=0.79; Figure 1). In particular, Observer 1 indicated retrospectively surgical treatment according to CI in 90 patients (40%), whilst Observer 2 indicated it in 81 (36%); between the two measurements a high agreement level (agreement 86.4%; expected agreement 49.9%; Kappa=0.72; z=7.44; p<0.0001) emerged.

**Figure 2:**
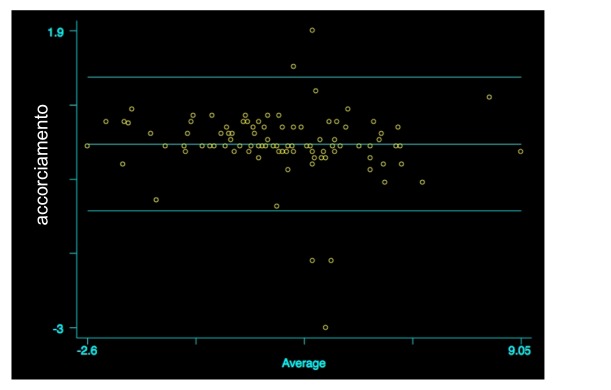
Comparison of radial shortening between the two measurements at T_0_, Bland Altman graph (r=-0.125; p=0.209).

At T_0_, the mean value of radial shortening, measured by Observer 1, was 2.6±2.1 (t=0.56; p=0.29) and 2.6±2.2, by Observer 2. Also in this case, the Bland Altman analysis did not give evidence of significant differences between the two measurements (r=-0.125; p=0.209; Figure 2).

**Figure 3:**
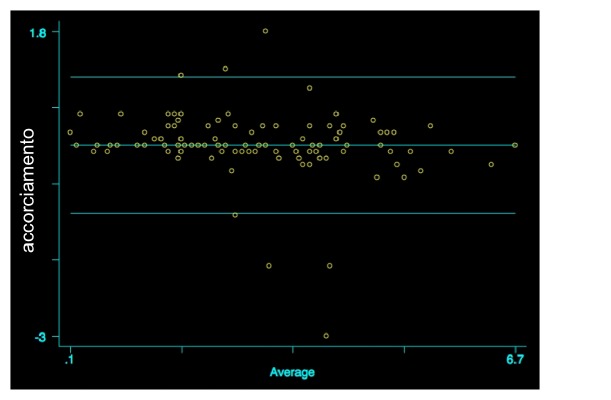
Comparison of radial shortening values measured by the two observers at T_1_, Bland Altman graph (r=-0.19; p=0.06).

At T_1_, the mean values of radial shortening measured by the two observers were, respectively, 2.8±1.4 and 2.8±1.5 (t=0.16; p=0.43). There were not any statistically significant differences (r=-0.19; p=0.06; Figure 3).

As regards pathological ranges compatible with functional or anatomical deficits, these were found in 18 patients (8%) according to Observer 1 and 27 (12%) according to Observer 2. The agreement level between the two evaluations was high (agreement 97.1%; expected agreement 86.4%; K=0.78; z=8.16; p<0.0001).

**Figure 4:**
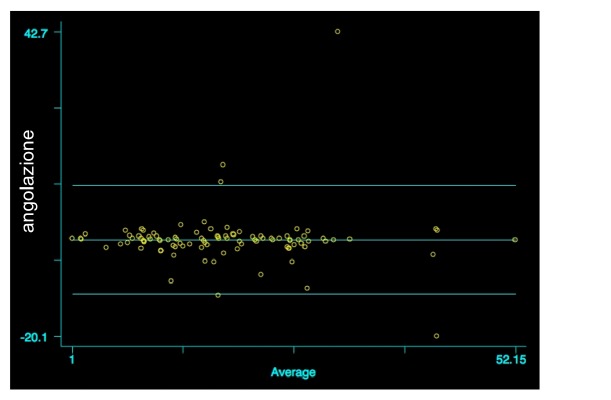
Comparison of angular values between the two measurements at T_0_, Bland Altman graph (r=-0.0004; p=0.964).

At T_0_, the mean angular value, measured by Observer 1, was 18.6±10.0 whilst for Observer 2 it was 18.9±10.0 (t=0.16; p=0.44). Also, in this case, Bland Altman analysis did not show any significant differences between the two measurements (r=-0.004; p=0.964; Figure 4).

**Figure 5:**
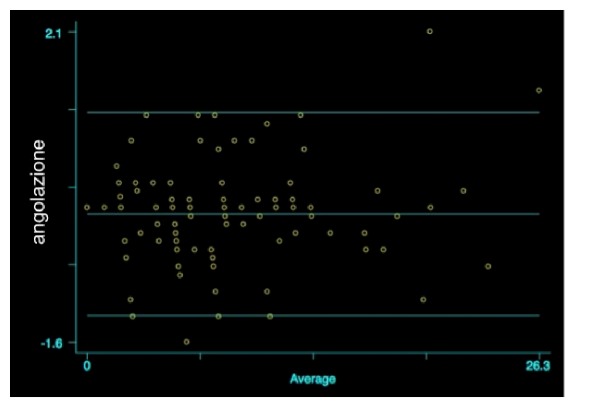
Comparison of angular values between the two measurements at T_1_, Bland Altman graph (r=0.041; p=0.678).

At T_1_ the angular mean values, measured by the two observers, were 7.7±5.8 for Observer 1 and 7.8±5.8 for Observer 2 (t=1.18; p=0.12). Also, in this case, the authors did not reveal any statistically significant differences (r=0.041; p=0.678; Figure 5).

The percentage of failures according to angular value at T_1_, was 26.7% (n=60) for Observer 1 and 22.7% (n=51) for Observer 2; the agreement level in this evaluation was very high (agreement: 99%; expected agreement: 69.3%; K=0.97; z=9.83; p<0.0001).

Analyzing the agreement between CI and radial translation with reference to the surgical indication, there was statistical significance for the measurements performed by Observer 2 (agreement 59.2%; expected agreement: 54.7%; k=0.01; p=0.025), whilst no statistically significant differences in the comparison between the measurements calculated by Observer 1 emerged (agreement 50.5%; expected agreement 49.5%; k=0.02; p=0.16).

Observer 1 indicated retrospectively surgical treatment in 90 (40%) of the 225 enrolled patients according to CI values. Observer 2 indicated retrospectively surgical treatment in 81 (36%) of the 225 enrolled patients.

As regards radial translation parameter, Observer 1 did not indicate retrospectively the surgical treatment, whilst Observer 2 indicated retrospectively the surgical treatment only in 3 cases out of 225.

In the study group the two observers reported a poor radiographic result in 78 patients (34.7%) according to shortening and angulation measured at T_1_.

Analyzing the CI measured by Observer 1, 90 patients out of 225 had retrospective indication to surgical treatment, whilst the remaining 135 received a conservative treatment indication. The frequency of failure was 18/135 (13.3%) for patients with a conservative treatment indication and 69/90 (66.6%) for patients with surgical treatment indication (chi-square=22.6; p<0.0001). The same analysis was carried out by Observer 2 and he indicated retrospectively the surgical treatment in 81/225. The proportion of failure was 21/144(14.6%) for the patients with retrospective conservative treatment indication and 57/81 (70.4%) for the patients with surgical treatment indication (chi-square=23.7 p<0.0001).

Furthermore, analyzing age groups, the proportion of patients with a retrospective surgical treatment indication characterized by poor results is described in Table 1.

As regards radial translation parameter, Table 2 describes the distribution for class assigned by the two observers. Observer 1 did not report any retrospective surgical indications in patients enrolled. The frequency of failure was 78/225 (34,7%) for patients with conservative treatment indication, whilst Observer 2 reported only 3/225 patients with retrospective surgical indication and those who had failure. The proportion of failure was 75/222 (33,8%) for the patients with retrospective indication to conservative treatment.

Moreover, as regards Observer 1, 78 patients with a poor result received a retrospective indication to conservative treatment according to radial translation; for the second observer the proportion was 75/222.

The results of measurements revealed at T_1_ of both affected and unaffected arm are described in Table 3. The mean value of the difference of pronation range between affected and unaffected arm in the patients with poor result was 4.3°±1.4° (range 0-9); in particular 48 out of the 78 patients who had failure (61.5%) presented a difference of pronation range >5°.

In these patients the mean value of the difference of supination range was 4.3°±1.3° (range: 3°-10°); in detail 42 out of the 78 patients who had failure (53.9%) presented a difference of supination range >5°.

## 4. Discussion

The authors evaluated the radiographic data of 225 patients, affected by forearm fractures, who had been treated conservatively; first they defined retrospectively the treatment indication according to CI and radial translation parameters. Then they revealed the poor results according to shortening and angulation parameters measured at T_1_ in the patients with retrospectively conservative indication. In this second step the poor radiographic results were linked to clinical-functional data.

To define the efficacy of radiographic parameters in terms of predictive conservative treatment failure, the measurements performed by the two observers with an experience greater than 10 years were analyzed statistically at T_0_ and T_1_.

As regards the first parameter (CI), the comparison of the mean values of measurements performed by the two observers did not evidence any statistically significant differences (r=0.027; p=0.79). Indeed, there was a high agreement level (agreement 86.4%).

As regards shortening, analyzing the values observed at T_0_ and T_1_, no statistically significant differences between the two measurements (T_0_: r=-0.125; p=0.209) (T_1_: r=-0.19; p=0.06) emerged. The reliability of the measurements performed by the two observers was very high as demonstrated by agreement level of 95.1%.

In relation to the angulation, the Bland Altman analysis did not evidence any statistically significant differences between T_0_ and T_1_ (T_0_: r=-0.004; p=0.964) (T_1_: r=0.041; p=0.678). Even though the evaluation of Figure 5 and the dispersion of values may appear contradictory, indeed, if we analyze in depth the dispersion grade, we may note that the range of values is very small, being in the order of up to 0.5° with a very high agreement level (agreement: 99%). The percentage of patients with pathological angular values was 26.7% for Observer 1 and 22.7% for Observer 2; we related the high agreement between the measurements to the accuracy detection of angular value with respect to the linear values which are influenced by variables of X-ray execution. In literature numerous works reported the measurements errors due to the variability of distance between the patient and X-ray tube, i.e., magnification concept and right radiographic position [19].

As regards radial translation a high agreement level emerged as demonstrated in Table 2. This was linked to the easiness of identification so to assign the class. Indeed, over 76% of patients presented a translation of distal segment and so were assigned to class 1.

In the last few years, though surgical treatment of forearm fracture, in particular biosseous, has become more frequent [12, 20, 21], conservative treatment is still by far the most common approach [21].

According to recent data of bibliography, also in our experience we verified a predominance of conservative treatment with respect to surgery.

For this reason, the predictive factors failure of conservative treatment plays the main role in recent literature taking into consideration that the causes of loss of reduction in forearm pediatric fractures are related to bone injury pattern [6], to the surgeon and to the patient [9].

Among the predictive failure factors linked to the pattern of injury, we reported the angulation and radial translation [15, 17], whilst among those related to surgeon and/or patient we highlighted the role of CI, padding index and Canterbury index [9].

Our study is the first in literature that compares two different parameters (CI and radial translation) in order to report the superiority in terms of reliability of each one or both of them in predicting failure.

As pointed out by the authors, the failure risk, in terms of angulation and shortening after reduction manoeuvre, is very low (about 7%) [22, 23].

The angulation parameter value changes according to the age [24, 25]. The value greater than 15° in the child aged <10 and 10° for the child aged >10 may affect aesthetic and/or pronosupination defects.

Furthermore, a radial shortening greater than 5 mm with respect to the ulna is not tolerated and causes pronosupination defects [16].

In the observation group, the authors revealed a percentage of failure equal to 34.7% according to the parameters (angulation and radial shortening described previously).

As defined by results, the authors observed a high agreement level between CI and radial translation for the measurements performed by both observers.

The analysis of CI, measured by Observer 1, showed that among the 135 patients out of 225 with a conservative retrospective indication (CI<0.84), 18 subjects presented a failure of reduction manoeuvre (13.3%). In the remaining 90 patients a value that indicated high risk for conservative treatment failure emerged (CI >0.84); in fact, 60 out of 90 patients had failure (66.6%) (chi-square = 22.6; p<0.0001).

The same analysis was carried out by Observer 2 who reported that 144 out of 225 patients had CI <0.84, and 21 of these had failure (14.6%). Similarly 57 out of 81 with CI that indicated the need for surgery (70.4%) had failure (chi-square = 23.7; p<0.0001).

As regards radial translation parameter, a 33.8% failure was observed in subjects whose retrospective indication was conservative treatment. This parameter therefore proved to be statistically less reliable than the CI. Indeed CI, the radiological parameter that regards both the surgeon and the patient, reflects the skill in applying the cast.

As reported in literature the respect of the principle of three-point system appears fundamental [14]. Another factor responsible for incorrect application of the cast is dependent on the age of the patients since in children >10 the increase of muscle mass means greater difficulty in modelling the cast [26]. In our experience, the analyses of different age groups for the patients with CI >0.84 and poor results showed failure increased with age. In the measurements performed by both observers in children >10 years, the failure rate was 100%. In fact, with an increase in age, we observed an increase of retrospective indication for surgery according to CI due to an increase in muscle mass and so also to the radiographic parameters.

The second aim of this study was to link the radiographic failure to clinical data. As described in Table 3, the authors verified a close link between the radiographic and functional results. As regards pronation deficit, a >5° mean value deficit of the pronation range with respect to unaffected arm was observed in 61.5% of the patients considered to have failure. As regards supination a >5° mean value deficit of the supination range with respect to unaffected arm was observed in 53.9% of the patients considered to have failure. In literature, the minimal value to define a pronosupination deficit must be ≥5° considering possible errors related to precision of the instruments and objectivity of the surgeon [27]. The authors demonstrated a functional deficit of pronosupination more than 50% of the patients undergoing conservative treatment. Based on the results obtained, we observed the superiority, in terms of reliability, of the CI over radial translation; this could be linked to the greater accuracy of CI which measures two planes of both lateral and anteroposterior view.

Radial translation, however, measured only in anteroposterior view, is probably less accurate notwithstanding the four classes. Regarding the latter, the division into two classes (classes II and III) may not be sufficient.

Moreover, according to the data of our study, in the evaluation of conservative treatment option in cases of discordance between the two parameters, we suggest relying on the CI.

At the same time, as defined in the literature, given that the causes of loss of reduction may be linked to different variables, it is better not to analyze just one radiographic parameter but to evaluate also the fractures parameters [3, 28].

The weak point of our study is the lack of analysis of other accurate radiographic parameters such as three-point index and gap index. In a work in progress, the authors will study the other radiographic parameters comparing the results and revealing the predictive efficacy of the respective parameters.

## 5. Conclusions

The achievement of our initial objectives allowed us to define the greater reliability of CI with respect to the radial translation parameter and the direct relationship between radiographic failure and clinical-functional data. In the evaluation of reduction manoeuvre failure and conservative treatment of forearm pediatric fracture, in cases where there is a discordance, the CI is the much more reliable parameter. Moreover, we should not rely on just one predictive factor but should take into account multifactor analysis. It is opportune, therefore, to consider the characteristics of the fracture, the morphology of the patient, and the surgeon.

## Figures and Tables

**Table 1:** The proportion of patients according to age groups and poor result who received a retrospective indication to surgical treatment in relation to CI.

Age Groups	Observer 1	Observer 2
<5 years	9/21 (42.8%)	9/21 (42.8%)
5-10 years	15/33 (45.4%)	15/27 (55.5%)
>10 years	36/36 (100%)	33/33(100%)

**Table 2:** Distribution of patients analyzed for radial translation class measured by the two observers and agreement level.

Radial translation class	Observer 1	Observer 2	Agreement	Expected agreement	Z	P
1	177 (78.7%)	171 (76%)	94.2%	56.4%	8.8	<0.001
2	48 (21.3%)	51 (22.7%)	93.2%	59.1%	8.5	<0.001
3	0	0	97.1%	95.2%	5.0	<0.001
4	0	3 (1.3%)	98.1%	98.1%	NA	NS

**Table 3:** Description of ROM values of both the affected and unaffected arm measured at T_1_.

	Range of Motion
AFFECTED ARM PRONATION	75.45° (66°-85°)
UNAFFECTED ARM PRONATION	79.90° (72°-88°)
AFFECTED ARM SUPINATION	75.44° (66°-85°)
UNAFFECTED ARM SUPINATION	79.89° (71°-88°)

## Data Availability

Readers are encouraged to contact the authors for details about the study population.
